# Curcumin Analogue L48H37 Suppresses Human Osteosarcoma U2OS and MG-63 Cells’ Migration and Invasion in Culture by Inhibition of uPA via the JAK/STAT Signaling Pathway

**DOI:** 10.3390/molecules26010030

**Published:** 2020-12-23

**Authors:** Ko-Hsiu Lu, Heng-Hsiung Wu, Renn-Chia Lin, Ya-Chiu Lin, Peace Wun-Ang Lu, Shun-Fa Yang, Jia-Sin Yang

**Affiliations:** 1 Department of Orthopedics, Chung Shan Medical University Hospital, Taichung 402, Taiwan; cshy307@csh.org.tw (K.-H.L.); cshy594@csh.org.tw (R.-C.L.); 2 School of Medicine, Chung Shan Medical University, Taichung 402, Taiwan; 3 Graduate Institute of Biomedical Science, China Medical University, Taichung 404, Taiwan; henghsiungwu@mail.cmu.edu.tw; 4 Research Center of Tumor Medical Science, China Medical University, Taichung 404, Taiwan; 5 Center for Molecular Medicine, China Medical University Hospital, Taichung 404, Taiwan; 6 Division of Hyperbaric Oxygen Therapy and Wound Medicine, Chung Shan Medical University Hospital, Taichung 402, Taiwan; 7 Institute of Medicine, Chung Shan Medical University, Taichung 402, Taiwan; champion-one@hotmail.com; 8 Morrison Academy Taichung, Taichung 406, Taiwan; lup@mca.org.tw; 9 Department of Medical Research, Chung Shan Medical University Hospital, Taichung 402, Taiwan

**Keywords:** L48H37, migration, osteosarcoma, JAK/STAT, uPA

## Abstract

Osteosarcoma, the most prevalent malignant bone tumor in the pediatric age group, is responsible for the great majority of cancer-associated deaths owing to its highly metastatic potential. The anti-metastatic effects of the new curcumin analogue L48H37 in human osteosarcoma are still unknown; hence, we investigated whether L48H37 represses human osteosarcoma cells’ biological behavior of migratory potential and invasive activities and attempted to delve into its underlying mechanisms. L48H37 up to 5 μM inhibited, without cytotoxicity, the motility, migration, and invasion of human osteosarcoma U2OS and MG-63 cells. In U2OS cells, the human protease array revealed an obvious decrease in urokinase plasminogen activator (uPA) expression after L48H37 treatment, and L48H37 actually reduced the level, protein and mRNA expression, and promoter activity of uPA dose-dependently. L48H37 decreased the phosphorylation of STAT3, JAK1, JAK2, and JAK3 in U2OS cells, but did not affect the phosphorylation of ERK, JNK, p38, and Akt. Using colivelin, an activator of STAT3, the L48H37-induced decrease in uPA and migratory potential could be countered as expected. Collectively, L48H37 represses the invasion and migration capabilities of U2OS and MG-63 cells by the suppression of uPA expression and the inhibition of JAK/STAT signaling. These results suggest that L48H37 may be a potential candidate for anti-metastatic treatment of human osteosarcoma.

## 1. Introduction

Osteosarcoma, mainly arising from the metaphysis of the distal femur or proximal tibia, is the most prevalent bone malignancy, with peak incidence in the second decade of life and the second incidence peak in older adulthood [1,2,3]. While the evaluation of osteosarcoma patients should include plain radiographs and magnetic resonance imaging of the entire affected bone, computed tomography of the chest and bone scan are needed for metastatic disease [1,4]. As a potent metastatic tumor, a cure is rare after surgical en bloc resection or amputation of the extensively diseased extremity to achieve a complete radical excision, which has been the treatment of choice for osteosarcoma [4,5]. Based on radiological staging, the combination of limb-sparing surgery and effective chemotherapy for osteosarcoma has increased long-term survival rates to approximately 68% recently [5,6]. However, early metastatic transfer to the lungs is still responsible for most treatment failure and is accountable for one of the most lethal pediatric malignancies [7].

Cancer metastasis involves highly coordinated, sequential, and complex pathways that are collectively termed the metastasis cascade [8], including the detachment of malignant cells, epithelial-mesenchymal transition (EMT), invasion and migration, intravasation, travel through lymph fluid and the bloodstream, extravasation, and reestablishment of growth at a distant site [2,9]. After the EMT of cancer cells, invasion of the basement membrane proceeds through a series of discrete steps, and various proteases predominantly control the degradation of the extracellular matrix (ECM) and the basement membrane of blood and lymph vessels [10]. Of these proteases, urokinase-type plasminogen activator (uPA), matrix metalloproteinase (MMP)-2 (gelatinase A, 72 kDa), and MMP-9 (gelatinase B, 92 kDa) are considered the most crucial enzymes for controlling the degradation of the main constituent of the ECM and are substantially involved in cancer invasion, migration, and metastasis [11,12]. Thus, suppressing uPA-, MMP-2-, or MMP-9-mediated cellular invasion and migration may generate a putative anti-metastasis effect.

Identifying molecular signaling pathways implicated in metastatic osteosarcoma contributes to the development of new therapeutic strategies available for patients who either metastasize or do not [2,3]. Several pathways, including mitogen-activated protein kinases (MAPKs), a family of serine/threonine kinases, including extracellular signal–regulated kinase (ERK) 1/2, c-Jun N-terminal kinase (JNK) 1/2 and p38, phosphoinositde 3-kinase (PI3K)/Akt, nuclear factor κB (NF-κB), and Janus kinase (JAK)/signal transducer and activator of transcription 3 (STAT3), are known to participate in various signaling cascades that are involved in the many cellular processes of motility, migration, invasion, and adhesion in osteosarcoma [13,14,15,16]. Therefore, activation of MAPKs, PI3K/Akt, JAK/STAT, and NF-κB signaling plays an important regulatory role in numerous cellular activities of osteosarcoma metastasis.

**Figure 1:**
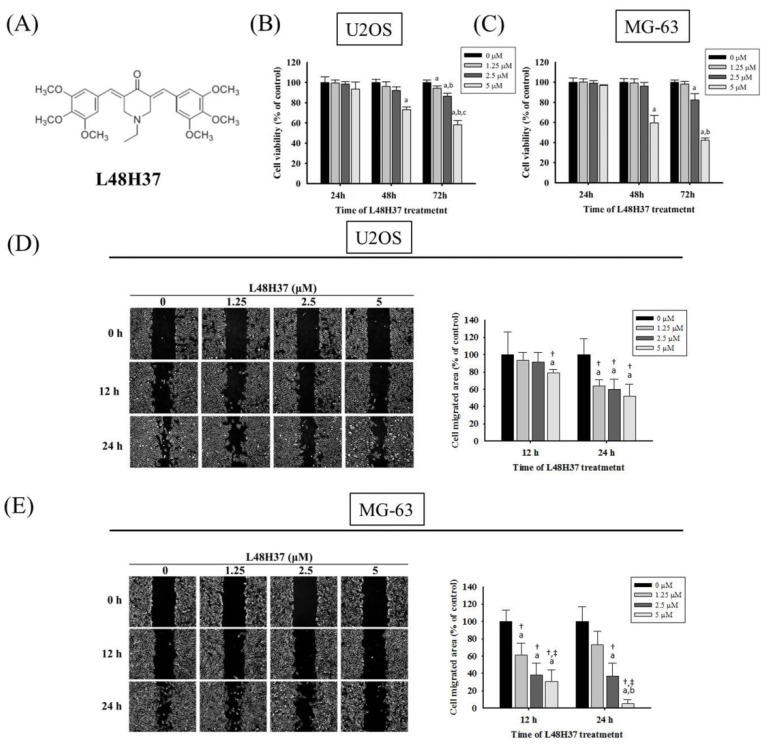
Effects of L48H37 on the viability of U2OS and MG-63 cells. (**A**) The structure of curcumin analogue L48H37. (**B**,**C**) Using an microculture tetrazolium (MTT) assay, the effects of L48H37 on the viability of U2OS and MG-63 cells treated with L48H37 (0, 1.25, 2.5, and 5 μM) for 24, 48, and 72 h were detected and illustrated after quantitative analysis. (**D**,**E**) The wound-healing assay after different concentrations (0, 1.25, 2.5, and 5 μM) of L48H37 treatment for different time intervals (0, 12, and 24 h) in U2OS and MG-63 cells were measured, as described in the Materials and Methods section, and illustrated after quantitative analysis. *n* = 3. ANOVA with Tukey’s post-hoc test was used. Concentration effects: U2OS: 24 h: F = 7.533, *p* = 0.010; MG-63: 12 h: F = 16.333, *p* = 0.001; 24 h: F = 26.228, *p* < 0.001. ^a^ Significantly different, *p* < 0.05, compared with the vehicle group. ^b^ Significantly different, *p* < 0.05, compared with 1.25 μM. ^c^ Significantly different, *p* < 0.05, compared with 2.5 μM. ^†^ Significantly different, *p* < 0.05, compared with 0 h. ^‡^ Significantly different, *p* < 0.05, when compared with 24 h (U2OS) or 12 h (MG-63).

Curcumin, a phenolic compound from the natural spice turmeric (*Curcuma longa*) possesses pleiotropic pharmacological effects, including anti-inflammatory [17], anti-oxidative [18], cardiovascular protective, and anti-cancer effects [19,20,21,22]. Poor absorption, quick metabolism, and rapid systemic elimination contribute to the low plasma and tissue levels of curcumin, and therefore limit its application in anti-cancer therapy. In an effort to increase the bioavailability and pharmacokinetic profiles of curcumin, several researchers have designed and synthesized potent anti-neoplastic analogues of curcumin [23]. L48H37 [1-ethyl-3,5-bis((*E*)-3,4,5-trimethoxybenzylidene)piperidin-4-one], a new curcumin analogue with an unsaturated monoketone structure compared to the β-diketone structure of curcumin, was developed to increase its stability and improve its bioavailability and anti-cancer effects (Figure 1A). So far, only two studies have demonstrated the anti-cancer effects of L48H37 in lung cancer [24] and pancreatic ductal adenocarcinoma cells [25]. However, the effect of L48H37 on human osteosarcoma metastasis remains to be fully delineated. We herein tested the hypothesis that L48H37 affects the invasion and migration of human osteosarcoma cells and explored its underlying pathways.

## 2. Results

### 2.1. Cytotoxicity of L48H37 in Osteosarcoma U2OS and MG-63 Cells

The cell viability of osteosarcoma U2OS and MG-63 cells in the presence of L48H37 with concentrations of 1.25, 2.5, and 5 μM for 24 h was not significantly different from that of the controls (0 μM) in the microculture tetrazolium (MTT) assay (U2OS: *p* = 0.87; MG-63: *p* = 0.382) (Figure 1B,C). However, after 72 h of treatment, the viability of U2OS and MG-63 cells in the presence of L48H37 at concentrations of 2.5 and 5 μM was significantly different from that of the control (0 μM) (Figure 1B,C) (*p* < 0.001 and *p* < 0.001). Thus, 24 h treatment with L48H37 up to 5 μM had no cytotoxic effect on U2OS and MG-63 cells. We used this concentration range for L48H37 in all subsequent experiments to explore its anti-metastatic properties.

### 2.2. L48H37 Represses U2OS and MG-63 Cells’ Motility, Migration, and Invasion

**Figure 2:**
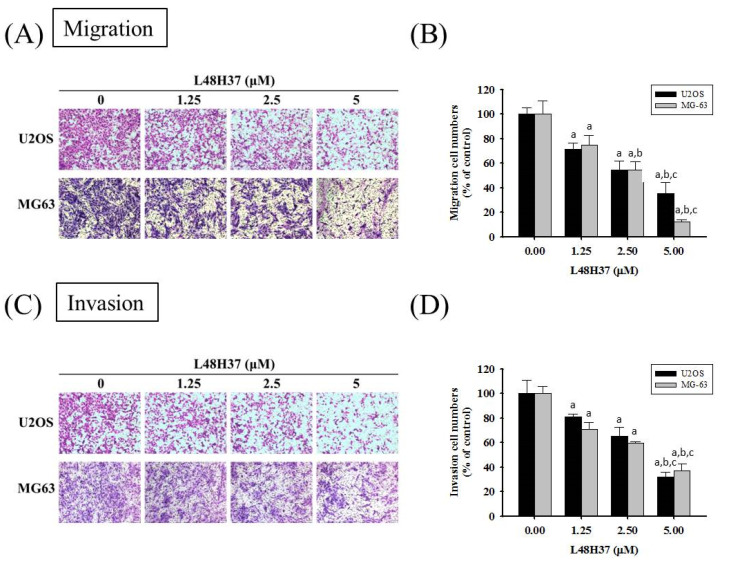
Effects of L48H37 on the in vitro cellular migration and invasion of U2OS and MG-63 cells. Cell migration (**A**,**B**) and invasion (**C**,**D**) assays after various concentrations (0, 1.25, 2.5, and 5 μM) of L48H37 treatment for 24 h in U2OS and 48 h in MG-63 cells were measured, as described in the Materials and Methods section, and illustrated after quantitative analysis. Results are shown as mean ± SD. *N* = 3. ANOVA with Tukey’s post-hoc test was used. Migration: U2OS: F = 50.518, *p* < 0.001; MG-63: F = 70.589, *p* < 0.001. Invasion: U2OS: F = 51.441, *p* < 0.001; MG-63: F = 83.112, *p* < 0.001. ^a^ Significantly different, *p* < 0.05, compared with control. ^b^ Significantly different, *p* < 0.05, compared with 1.25 μM. ^c^ Significantly different, *p* < 0.05, compared with 2.5 μM.

After treatment for 24 h in U2OS cells and MG-63 cells, L48H37 significantly reduced the motility of U2OS and MG-63 cells in the wound-healing assay (concentration effects: U2OS: 24 h: *p* = 0.010; MG-63: 12 h: *p* = 0.001, 24 h: *p* < 0.001 (Figure 1D,E). Moreover, the Boyden chamber assay with and without Matrigel, after treatment for 24 h in U2OS cells and 48 h in MG-63 cells, showed that L48H37 significantly and dose-dependently reduced the migratory potential and invasive activity in U2OS and MG-63 cells (migration: U2OS: *p* < 0.001, MG-63: *p* < 0.001; invasion: U2OS: *p* < 0.001; MG-63: *p* < 0.001) (Figure 2A–D).

### 2.3. L48H37 Reduces the Level, Protein and mRNA Expression, and Promoter Activity of uPA in U2OS Cells

**Figure 3:**
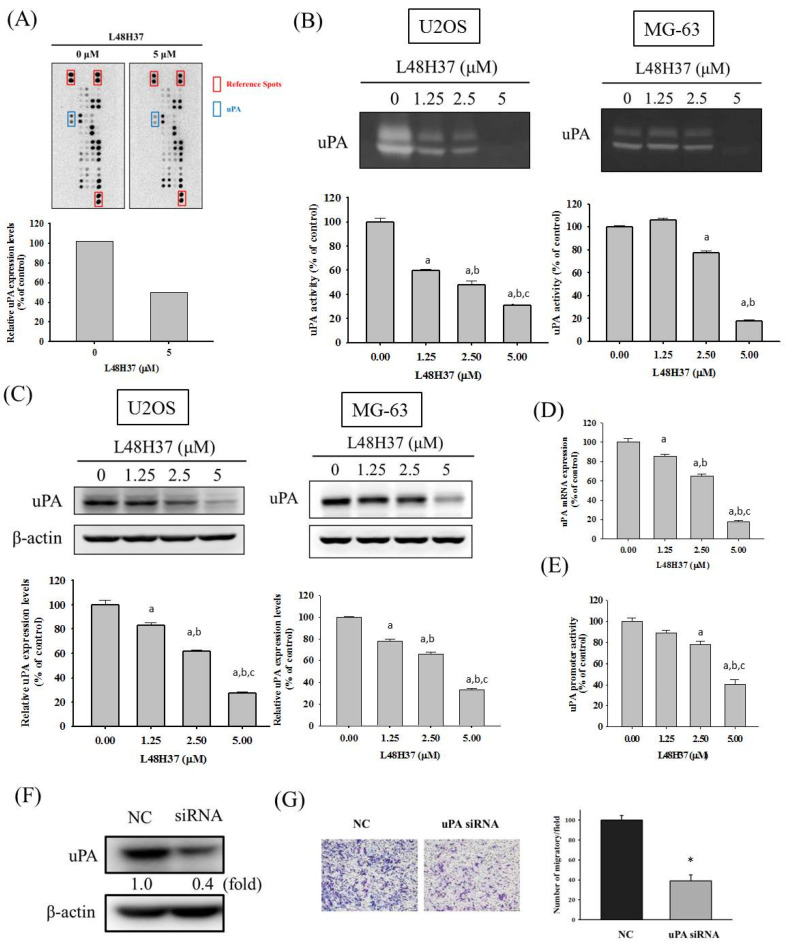
Expression of uPA in L48H37-treated U2OS cells. (**A**) The protease array, after treatment with 5 μM of L48H37 for 24 h in U2OS cells, was employed, as described in the Materials and Methods section, and illustrated after quantitative analysis. (**B**) Casein zymography, (**C**) Western blotting analysis, (**D**) real-time PCR, and (**E**) luciferase reporter assay, after treatment with L48H37 at various concentrations (0, 1.25, 2.5, and 5 μM) for 24 h in U2OS cells were conducted as described in the Materials and Methods section. Results are shown as mean ± SD. *n* = 3. ANOVA with Tukey’s post-hoc test was used. ^a^ Significantly different, *p* < 0.05, compared with control. ^b^ Significantly different, *p* < 0.05, compared with 1.25 μM. ^c^ Significantly different, *p* < 0.05, compared with 2.5 μM. (**F**) Western blotting assay to confirm siRNA directly against uPA expression and (**G**) Boyden chamber assay after treatment of uPA siRNA for 24 h in U2OS cells were conducted, and the effects were illustrated after quantitative analysis. Student’s *t*-test was used. ^*^ Significantly different, *p* < 0.05 compared with the negative control (NC) group.

To identify the underlying mechanism of the anti-metastatic actions of L48H37 in osteosarcoma cells, we employed the protease array, which showed repression of uPA expression in U2OS cells after treatment with 5 μM of L48H37 for 24 h (Figure 3A). We subsequently conducted casein zymography and Western blotting assay to validate the finding of the protease array and found that L48H37 significantly repressed the uPA level and protein expression in the U2OS and MG-63 cells (U2OS: level: *p* < 0.001; protein: *p* < 0.001; MG-63: level: *p* < 0.001; protein: *p* < 0.001) (Figure 3B,C). We also conducted real-time PCR and luciferase reporter assays and found that L48H37 significantly repressed the mRNA expression and promoter activity of uPA in U2OS cells (mRNA: *p* < 0.001; promoter activity: *p* < 0.001) (Figure 3D,E). We transformed cells with a small interfering RNA (siRNA)-targeting uPA expression for 24 h and measured the uPA protein expression in Western blotting to further confirm whether reduction in uPA interferes with the migratory activity of U2OS cells (Figure 3F). Subsequently, we performed Boyden chamber migration while using the siRNA of uPA for 24 h to compare the migratory ability and found that the knockdown of uPA significantly decreased the migratory potential of U2OS cells (*p* < 0.001) (Figure 3G).

### 2.4. L48H37 Suppresses the JAK/STAT Pathway in U2OS Cells

**Figure 4:**
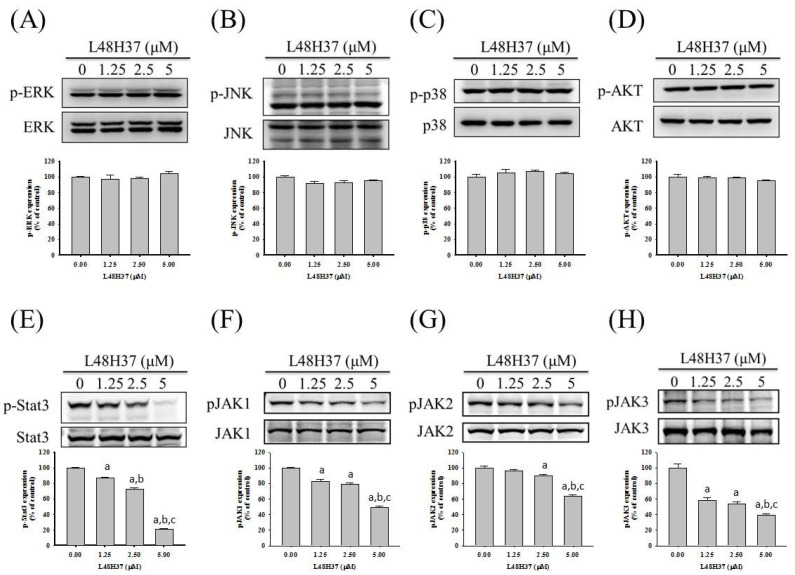
Effects of L48H37 on MAPKs, PI3K-Akt, STAT3, and JAK1/2/3 in U2OS cells. Western blotting analyses for the total or phosphorylated forms of (**A**) ERK, (**B**) JNK, (**C**) p38, (**D**) Akt, (**E**) STAT3, (**F**) JAK1, (**G**) JAK2, and (**H**) JAK3 after treatment with L48H37 at various concentrations (0, 1.25, 2.5, and 5 μM) for 24 h in U2OS cells were conducted as described in the Materials and Methods section. The effects were illustrated after quantitative analysis. Results are shown as mean ± SD. *n* = 3. ANOVA with Tukey’s post-hoc test was used. ERK: F = 0.820, *p* = 0.518; JNK: F = 2.986, *p* = 0.096; p38: F = 0.821, *p* = 0.518; Akt: F = 1.247, *p* = 0.355; STAT3: F = 1074.631, *p* < 0.001; JAK1: F = 140.865, *p* < 0.001; JNK2: F = 68.922, *p* < 0.001; JNK3: F = 67.086, *p* < 0.001. ^a^ Significantly different, *p* < 0.05, compared with control. ^b^ Significantly different, *p* < 0.05, compared with 1.25 μM. ^c^ Significantly different, *p* < 0.05, compared with 2.5 μM.

Since MAPK and PI3K pathways may be upstream signaling for uPA, Western blotting was employed to further investigate the molecular mechanisms. In the analysis, MAPK and PI3K/Akt pathways were detected in U2OS cells. As a result, however, L48H37 had no obvious influence on ERK 1/2, JNK 1/2, p38, or Akt, including their phosphorylation (ERK: *p* = 0.518, JNK: *p* = 0.096, p38: *p* = 0.518, Akt: *p* = 0.355) (Figure 4A–D). Furthermore, we explored the JAK/STAT pathway and intriguingly found that L48H37 decreased the phosphorylation of STAT3 and JAK 1/2/3 in U2OS cells (STAT3: *p* < 0.001, JAK1: *p* < 0.001, JNK2: *p* < 0.001, JNK3: *p* < 0.001) (Figure 4E–H).

### 2.5. L48H37 Inhibits Cellular Migration and uPA Expression via the JAK/STAT Pathway in U2OS Cells

**Figure 5:**
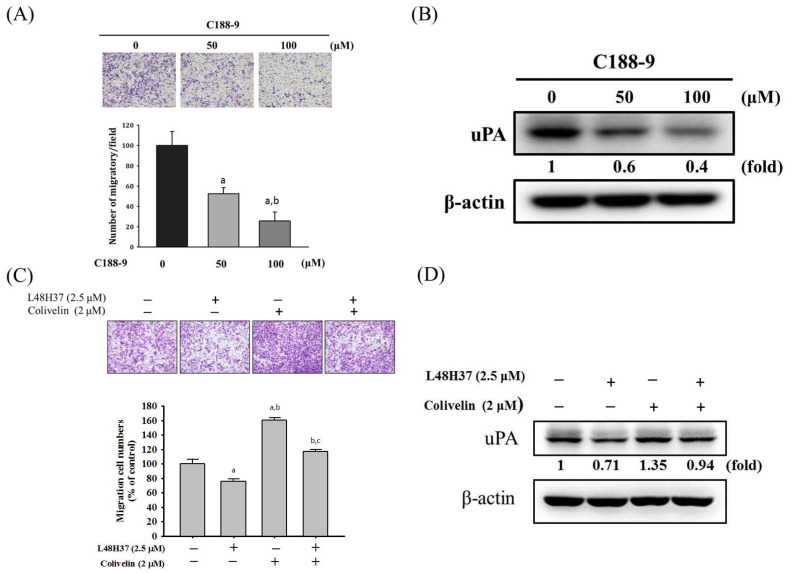
Effects of STAT3 inhibitor (C188-9) and activator (colivelin) on cell migration and uPA expression in L48H37-treated U2OS cells. (**A**) Migratory potential and (**B**) uPA expression after treatment of 50 and 100 μM of C188-9 for 24 h in U2OS cells were measured through Boyden chamber assays and Western blotting analysis. ^a^ Significantly different, *p* < 0.05, compared with control. ^b^ Significantly different, *p* < 0.05, compared with 50 μM of C188-9. (**C**) Migratory potential and (**D**) uPA expression after pretreatment with or without 2 μM of colivelin for 1 h, followed by treatment with or without 2.5 μM of L48H37 for an additional 24 h in U2OS cells, were measured through Boyden chamber assays and Western blotting analysis. The effects were illustrated after quantitative analysis. Results are shown as mean ± SD. *n* = 3. ANOVA with Tukey’s post-hoc test was used. Migration: F = 76.962, *p* < 0.001; uPA: F = 220.752, *p* < 0.001. ^a^ Significantly different, *p* < 0.05, compared with control. ^b^ Significantly different, *p* < 0.05, compared with 2.5 μM of L48H37. ^c^ Significantly different, *p* < 0.05, compared with 2 μM of colivelin.

To recognize whether the suppression of STAT3 and JAK 1/2/3 phosphorylation by L48H37 interferes with the actions of uPA secretion and migration potential by L48H37 in U2OS cells, an inhibitor of STAT3 (C188-9) was used. As shown in Figure 5A,B, cell migration potential and uPA expression in the U2OS cells were repressed by C188-9. We also used an activator of STAT3 (2 μM of colivelin) with or without treatment with 2.5 μM of L48H37 in U2OS cells. Migration potential and uPA expression were repressed by L48H37 (migration: *p* = 0.013) and activated by colivelin (migration: *p* < 0.001), as expected (Figure 5C,D). Intriguingly, colivelin significantly countered the decrease in migration potential and uPA expression in U2OS cells caused by L48H37 (migration: *p* < 0.001). Overall, these findings indicated that the JAK/STAT pathway plays a critical upstream role in L48H37-inhibited migration potential and uPA expression in U2OS cells.

## 3. Discussion

Although curcumin is a promising molecule to suppress the various metastatic properties of osteosarcoma cells [16,26], its low solubility in water and instability due to its chemical structure change result in poor bioavailability, about 75% of oral intake is excreted through the feces [27]. To effectively harness the anti-metastatic activity of curcumin, improve its pharmacokinetic properties and stability, enhance its ability for intravenous administration, and achieve prolonged half-lives, several curcumin analogues have been engineered [27]. The promising curcumin analogue L48H37 not only induces apoptosis through ROS-mediated endoplasmic reticulum stress and STAT3 pathways in human lung cancer cells [24] but also exerts a potent anti-cancer effect in human pancreatic ductal adenocarcinoma cells, which is augmented by histonelysine *N*-methyltransferase 2D [25].

In the present study, we investigated the anti-metastatic effects of L48H37 that can attenuate the cellular motility, migratory potential, and invasive ability of U2OS and MG-63 cells under a non-cytotoxic concentration range. Although MMP-2 and MMP-9 are well known to be key enzymes and contributed to the process of osteosarcoma cell metastases in our previous research [13,28,29,30], no effects of L48H37 on MMP-2 and MMP-9 secretion in U2OS cells were observed. Interestingly, repression of uPA protein expression in U2OS cells was detected after treatment with 5 μM of L48H37 in the protease array, and the L48H37-induced repression of uPA was verified using casein zymography, Western blotting, real-time PCR, and luciferase reporter assay. Through further analysis of upstream signaling, L48H37 decreased the phosphorylation of STAT3 and JAK 1/2/3 in U2OS cells, whereas there was no evident influence on ERK 1/2, JNK 1/2, p38, and Akt, or on their phosphorylation. The decrease in migratory potential and uPA expression in U2OS cells, caused by L48H37, was countered by activation of STAT3 colivelin. These results implied that L48H37′s inhibition of migration in human osteosarcoma U2OS cells results from the attenuation of uPA, not MMP-2 or MMP-9, through the JAK/STAT pathway rather than MAPK and PI3K/Akt signaling. However, in Figure 5C,D, a very marginal increase in uPA expression correlates with a much more striking increase in migratory potential after treatment with colivelin. These are indications that STAT activation likely activates other downstream effectors in addition to uPA and may warrant further studies.

The diverse MAPK members PI3K/Akt and STAT3 are activated in response to various extracellular stimuli and have distinct downstream targets, including cell motility, migration, invasion, proteinase induction, and angiogenesis, which all contribute to metastasis [31,32,33,34,35], so they have been implicated as oncogenes and therapeutic targets in a variety of neoplastic diseases. In human osteosarcoma SJSA and U2OS cell lines, a structure-based curcumin analogue, FLLL32, simultaneously decreases STAT3 DNA-binding activity, and expression of survivin, VEGF, and MMP-2 at both mRNA and protein levels, with concurrent decreases in phosphorylated and total STAT3, to promote loss of cell proliferation, leading to caspase-3-dependent apoptosis [36]. In MG-63 cells, curcumin dose-dependently decreases pJAK-2 and pSTAT-3 expression not only to induce cell cycle arrest and apoptosis but also to inhibit cellular migration and invasion [16]. As mentioned earlier, through ROS-mediated endoplasmic reticulum stress and STAT3 pathways to induce apoptosis in human lung cancer cells [24], L48H37 also repressed uPA via the JAK/STAT signaling to suppress the migration of human osteosarcoma U2OS cells in the present study.

## 4. Materials and Methods

### 4.1. Materials

Cell culture materials, including Dulbecco’s modified Eagle medium (DMEM) and fetal bovine serum (FBS), were obtained from Gibco Life Technologies (Gaithersburg, MD, USA) and Hyclone Laboratories, Inc. (Logan, UT, USA), respectively. Antibodies specifically for β-actin, Akt, and p38 were obtained from BD Biosciences (San Jose, CA, USA). Antibodies specific for phosphorylated ERK 1/2, JNK 1/2, Akt, STAT3, and JAK 1/2/3, as well as ERK 1/2, JNK 1/2, STAT3, and JAK 1/2/3, were purchased from Cell Signaling Technology (Danvers, MA, USA). Human uPA siRNA (s10610) and negative-control siRNA (4390844) were purchased from Applied Biosystems Instruments (Foster City, CA, USA). The Human Protease Assay Kit was acquired from R&D Systems (Minneapolis, MN, USA). L48H37 and the inhibitor of STAT3 (C188-9) were bought from Sigma-Aldrich (St. Louis, MO, USA). The activator of STAT3 (colivelin) was bought from Santa Cruz Biotechnology, Inc. (Dallas, TX, USA).

### 4.2. Cell Culture and L48H37 Treatment

Human osteosarcoma U2OS (15-year-old female) and MG-63 (14-year-old male) cells, obtained from the Food Industry Research and Development Institute (Hsinchu, Taiwan), were supplemented with 10% FBS, 1% penicillin/streptomycin, and 5 mL of glutamine, cultured in DMEM, and maintained at 37 °C in the humidified atmosphere of a 5% CO_2_ incubator, as described previously [14].

### 4.3. MTT Assay

To investigate the cytotoxicity of L48H37, the MTT colorimetric assay was conducted. We plated 8 × 10^4^/well U2OS and MG-63 cells in 24-well plates for 16 h and tested different concentrations (0, 1.25, 2.5, and 5 μM) of L48H37 at 37 °C for 24 h. After the exposure period, an MTT assay was carried out, as reported previously [32,37].

### 4.4. Wound-Healing Assay

For cellular motility, a total of 2.4 × 10^6^ U2OS cells and 1.5 × 10^6^ MG-63 cells in 6 cm dishes were plated for 16 h, wounded by scratching with a pipette tip, and then incubated with DMEM containing 0.5% FBS. We tested the indicated concentrations of L48H37 (0, 1.25, 2.5, and 5 μM) in U2OS cells for 0, 12, and 24 h, and MG-63 cells for 0, 12, and 24 h, respectively. The cells were photographed using a phase-contrast microscope (100×) to determine the width of the remaining wound area relative to the initial wound width, as depicted previously [30,37].

### 4.5. Cell Migration and Invasion Assays

To test the effect of L48H37 on the migratory potential and invasive ability of U2OS and MG-63 cells in vitro, we employed a modified Boyden chamber migration assay with and without Matrigel coating, respectively. After treatment with L48H37 with concentrations of 0, 1.25, 2.5, and 5 μM, the cells were seeded into the upper section of the Boyden chamber (Neuro Probe, Cabin John, MD, USA) at a density of 2 × 10^5^/mL for U2OS cells and 4 × 10^5^/mL for MG-63 cells, and then the U2OS cells and MG-63 cells were incubated in the modified Boyden chamber migration assay and invasion assay at 37 °C for 24 h and 48 h, respectively. Under a light microscope, the migratory and invasive cells were finally counted, as stated previously [32,37,38,39].

### 4.6. Protease Array Analysis

To determine metastasis-related proteins induced by L48H37, a protease (35 proteases) array analysis was employed to evaluate the protein lysates of 2 × 10^6^ U2OS cells from vehicle- or 5 μM L48H37-treated cells, according to the manufacturer’s protocols (R&D Systems, Minneapolis, MN, USA).

### 4.7. Preparation of Cell Lysates and Western Blotting Analysis

To validate the protease array findings and to further explore the molecular mechanism and signaling pathways, Western blotting analysis was conducted. We plated 8 × 10^5^ U2OS and MG-63 cells in 6 cm dishes for 16 h and tested them with different concentrations (0, 1.25, 2.5, and 5 μM) of L48H37 for 24 h. The total cell lysates of U2OS and MG-63 cells were prepared, as described previously [29,32,37]. Western blotting analysis was performed using either specific primary antibodies against three MAPKs (ERK 1/2, JNK 1/2, and p38), Akt, STAT3, and JAK 1/2/3 or with the specific antibodies for unphosphorylated or phosphorylated forms of the corresponding ERK 1/2, JNK 1/2, p38, Akt, STAT3, and JAK 1/2/3. As described previously, the blots were then incubated with a horseradish peroxidase goat anti-rabbit or anti-mouse IgG for 1 h, and the intensity of each band was measured by densitometry [29,32,37].

### 4.8. Real-Time Polymerase Chain Reaction (PCR)

With the following modification, a real-time PCR assay was performed, as described previously [32]. Specific primers and fluorogenic probes were used for the uPA gene.

### 4.9. uPA Promoter-Driven Luciferase Reporter Assay

We plated 8 × 10^4^/well U2OS cells in 6-well plates with treatments of 0, 1.25, 2.5, and 5 μM L48H37 for 24 h. As stated previously, the pGL3-basic vector (pGL3-control vector) and pGL3-uPA promoter were co-transfected with a β-galactosidase expression vector. After 24 h of transfection, the cell lysates were harvested and luciferase activity was determined using a luciferase assay kit. The value of the luciferase activity was normalized to transfection efficiency and monitored by β-galactosidase expression [37].

### 4.10. Statistical Analysis

For all of the measurements, analysis of variance was followed by one-way analysis of variance with Scheffe’s and Tukey’s post-hoc test for more than two groups with unequal and equal sample sizes per group, respectively. When two groups were compared, the data were analyzed while using Student’s t-test. Each experiment was repeated independently at least three times (*n* ≥ 3). *p* values < 0.05 were considered statistically significant.

## 5. Conclusions

In conclusion, the new curcumin analogue L48H37 contributes to the suppression of cellular invasion and migration by inhibition of uPA and the JAK/STAT pathway, not via ERK, JNK, p38, and PI3K/Akt signaling. This phenomenon of uPA repression of invasion and migration in U2OS and MG-63 cells could be activated by colivelin, an activator of STAT3. Our results reinforce the hypothesis that L48H37 possesses the ability to inhibit invasion and migration on human osteosarcoma cells, which provides evidence of being a potential candidate for anti-metastasis of human osteosarcoma, as well as a better understanding of the mechanisms responsible for these effects. In the future, the determination of the therapeutic efficacy and pharmacodynamics properties of L48H37 on osteosarcoma metastasis in vivo is imperative.

## Data Availability

The data presented in this study are available in this article.

## Sample Availability

Not available.
